# Effects of carbon-based nanomaterials on seed germination, biomass accumulation and salt stress response of bioenergy crops

**DOI:** 10.1371/journal.pone.0202274

**Published:** 2018-08-28

**Authors:** Kamal Pandey, Mohamed H. Lahiani, Victoria K. Hicks, M. Keith Hudson, Micah J. Green, Mariya Khodakovskaya

**Affiliations:** 1 Department of Biology, the University of Arkansas at Little Rock, Little Rock, Arkansas, United States of America; 2 Artie McFerrin Department of Chemical Engineering, Texas A&M University, College Station, Texas, United States of America; 3 Department of Chemistry, the University of Arkansas at Little Rock, Little Rock, Arkansas, United States of America; 4 Federal Scientific Center of the East Asia Terrestrial Biodiversity, Far-Eastern Branch of Russian Academy of Sciences, Vladivostok, Russia; University of Calcutta, INDIA

## Abstract

Bioenergy crops are an attractive option for use in energy production. A good plant candidate for bioenergy applications should produce a high amount of biomass and resist harsh environmental conditions. Carbon-based nanomaterials (CBNs) have been described as promising seed germination and plant growth regulators. In this paper, we tested the impact of two CBNs: graphene and multi-walled carbon nanotubes (CNTs) on germination and biomass production of two major bioenergy crops (sorghum and switchgrass). The application of graphene and CNTs increased the germination rate of switchgrass seeds and led to an early germination of sorghum seeds. The exposure of switchgrass to graphene (200 mg/l) resulted in a 28% increase of total biomass produced compared to untreated plants. We tested the impact of CBNs on bioenergy crops under salt stress conditions and discovered that CBNs can significantly reduce symptoms of salt stress imposed by the addition of NaCl into the growth medium. Using an ion selective electrode, we demonstrated that the concentration of Na^+^ ions in NaCl solution can be significantly decreased by the addition of CNTs to the salt solution. Our data confirmed the potential of CBNs as plant growth regulators for non-food crops and demonstrated the role of CBNs in the protection of plants against salt stress by desalination of saline growth medium.

## Introduction

The use of fossil fuels has accelerated since the dawn of the industrial revolution and demand will increase dramatically in response to an ever-increasing population and by a higher need for energy by mechanization [1]. It is reported that the energy demand will be increased by more than 50% due to rapid progress in all sectors including infrastructure development by the year 2025 [2]. However, the predominant fossil fuel power source is restricted [3]. Limited resources, controversy, environmental concerns and frequently increasing prices associated with the fossil fuels underscore the need to develop an alternative source of energy [4]. For this reason, scientists are looking to bioenergy as a complement to fossil fuels. The concept of bioenergy refers to new alternate renewable energy from biological materials that can generate heat, electricity and transportation fuels [5]. Any plant materials that are used to produce energy are referenced as bioenergy crops. Bioenergy crops are mainly cultivated for power generation including electricity, heat, and liquid fuels for transportation of motor vehicle [6]. Moreover, growing practice of bioenergy crops helps to reduce our dependence on existing fossil energy, reduce global warming by lowering the greenhouse gas, as well as creates job opportunities for thousands of people globally [7]. Bioenergy crops can be cultivated in marginal soils as an energy source due to their potential for a higher amount of biomass production [8] with a low requirement for fertilizers and irrigation. Currently, many countries (mainly Europe, USA, Brazil and Australia) have implemented policies to encourage energy production from plants. It is estimated that about 273–1381 EJ/energy is provided by bioenergy [9]. Early seed germination with higher germination rate, fast growth, and development, larger biomass yield, and tolerance to stresses are pivotal features of potential bioenergy crops [10].

**Fig 1:**
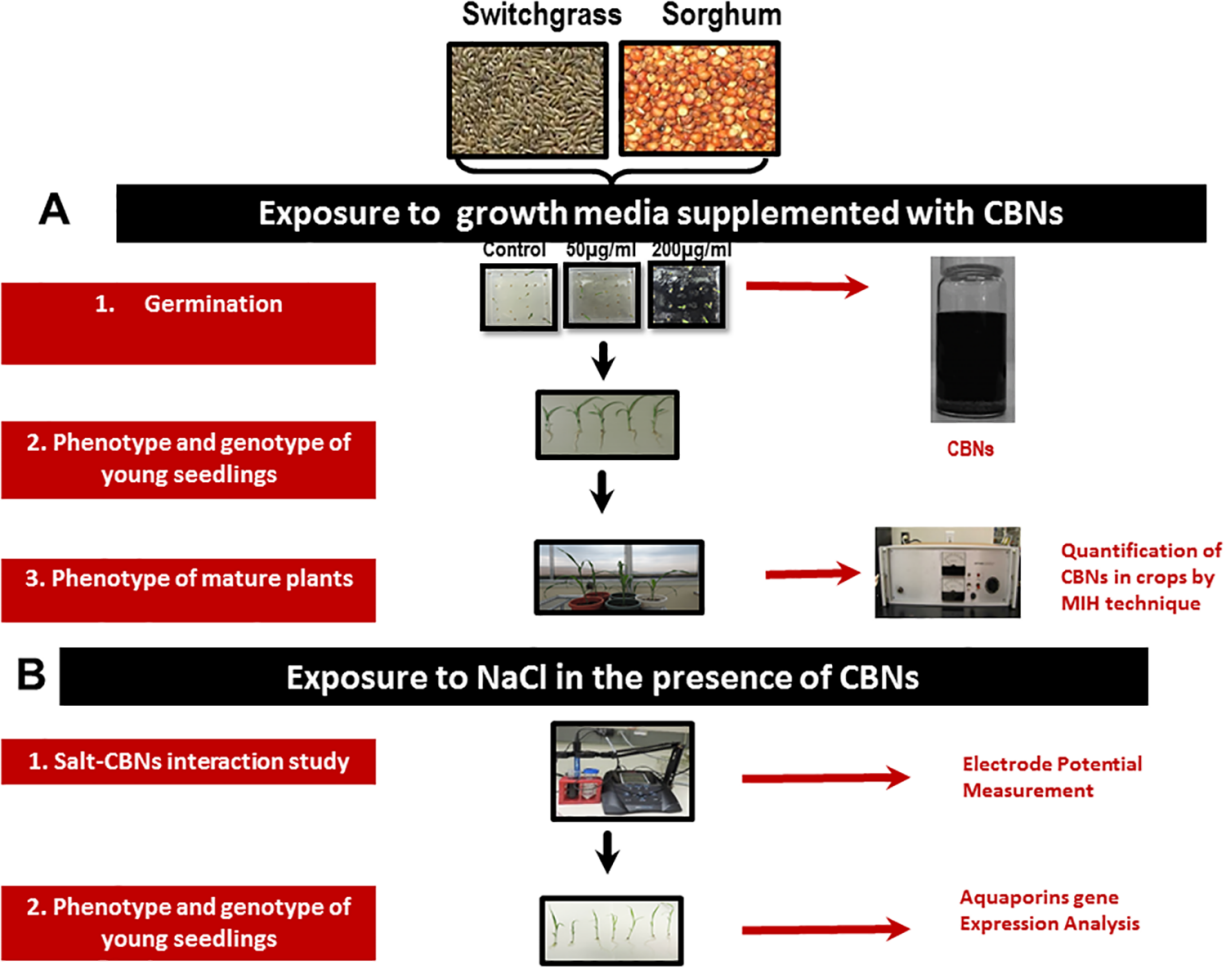
Schematic diagram showing the experimental design of this study. The effects of CBNs in seed germination, growth, and development of bioenergy crops. The seeds of sorghum and switchgrass were exposed to graphene or multi-walled CNTs by addition to the growth medium. Germination and plant growth between CBN-treated and control bioenergy crops were calculated. The quantification of multi-walled CNTs inside the shoots of matured bioenergy crops was performed using the microwave induced heating (MIH) technique. For salt stress experiments, seeds were exposed to growth medium supplimented with NaCl and different concentration of CNTs or graphene (50, 100, 200, 500, 1000 μg/ml) and germination and seedling growth was monitered. The physical interaction between multi-walled CNTs and ions (Na^+^ or Cl¯) presented in salty solutions supplemented with CNTs was confirmed by measuring the electrode potential using ion-selective electrodes.

The productivity of plants, including bioenergy crops, is limited by several critical factors such as genetic potential, biotic, abiotic, and nutritional stress. Thus, the search for new technologies that can lead to the enhancement of plant productivity is a constant task. It was demonstrated recently that certain nanomaterials may regulate productivity by the enhancement of plant growth [11]. Our laboratory discovered that a wide range of CBNs in low doses can activate seed germination [11], plant growth, and development of model plants as well as crop species such as barley, corn, and soybean [11–17]. The uptake and accumulation of CBNs in exposed plant tissues was confirmed using microscopy (TEM) and spectroscopy (Raman Spectroscopy) [12, 15, 17]. Recently, the exact concentration of CBNs absorbed by exposed plants including carbon nanotubes and carbon nanohorns was measured by the microwave induced heating (MIH) technique in different plant organs [16, 18]. The documented presence of nanomaterials used as plant growth regulators can be taken as an alarming sign of a possible transfer of CBNs in the food chain by consumption of crops contaminated with nanomaterials. Thus, the potential toxicity of CBN-contaminated food derived from agricultural crops exposed to CBNs has to be investigated experimentally. However, concern about the safety of use of CBNs for plant growth regulation can be less significant if nanomaterials will be applied to non-food plant species such as bioenergy crops which are not subject to food consumption. Here, we describe the efficiency of two types of CBNs, graphene and multi-walled CNTs, for regulation of seed germination and activation of biomass production of two different bioenergy crops *Sorghum bicolor* L. Moench and *Panicum virgatum* L. We also made an attempt to understand how the application of CBNs will affect the abiotic stress response of exposed bioenergy species. It was previously reported that carbon nanotube membranes are efficient for desalination of salty water [19, 20]. It is well known that salt stress is one of the major abiotic factors that limits sustainable crop production throughout the world [21]. A higher level of soil salinity limits seed germination as well as growth and development of plants [22]. Salt stress is responsible for the reduction of the tremendous amount of biomass accumulation of energy crops (*Miscanthus* × *giganteus*) [23]. Salinity not only adversely affects plant productivity but also quality. More than 20% of agricultural land is already damaged by salinization due to poor drainage system and irrigation of salty water [24]. Here, we demonstrated that CBNs (CNTs) added to growth medium can significantly reduce symptoms of salt stress in bioenergy crops exposed to salt stress by removal of toxic Na^+^ ions from salt solution. Fig 1 illustrates the experimental design for a study focused on the effects of CBNs on germination, growth and stress response of sorghum and switchgrass.

## Materials and methods

### Materials

Commercially available CBNs were used for our studies. Graphene nanoplatelets (<3 layers; lateral dimensions 1–2 μm) and multi-walled CNTs (MWCNT-COOH, OD 13–18 nm; length 1–12 μm) were purchased from Cheap Tubes (Brattleboro, VT). The graphene and CNTs were characterized using microscopy and spectroscopy techniques as was described in our previous paper [17]. The removal of endotoxins potentially presented in CBNs was performed by autoclavation as was previously described [25]. Seeds of sorghum (*Sorghum bicolor* L. Moench) and switchgrass (*Panicum virgatum* L.) were obtained from Sheffield’s Seeds Co. Inc., NY, USA.

#### Media preparation, seed germination, and seedling growth in vitro

The seeds of switchgrass and sorghum were sterilized by washing with 70% ethanol for two minutes and then washing with double distilled water, followed by 50% bleach and vortexed for about 30 minutes. Finally, seeds were washed nine times with sterile water. Murashige and Skoog (MS) medium [26] was used for germination of untreated (control) seeds and MS media supplemented with multi-walled CNTs (50 μg/ml and 200 μg/ml CNTs), and graphene (50 μg/ml and 200 μg/ml graphene) was used for the treatment of seeds with CBNs. The 60 sorghum seeds and 120 switchgrass seeds were inoculated on the control and each experimental medium under aseptic conditions (10 sorghum seeds/Magenta box and 20 switchgrass seeds/Magenta box) and incubated in the growth chamber (Percival Scientific, www.percival-scientific.com). During the incubation period, the chamber was maintained at 24°C, with 12 h-day photoperiods and a light intensity of 105 μmol/s m^2^. Seed germination rate was measured every day for 10 days for sorghum and 21 days for switchgrass. The phenotype of developed seedlings was recorded by measurements of root/shoot lengths and fresh/dry biomass weight. Experiments were repeated three times.

Considering the interactions of CBNs with salt [27], we investigated the response of bioenergy crops to salt stress in the presence of CBNs (graphene, CNTs). As a first step, we studied the sensitivity of sorghum and switchgrass to different concentrations of NaCl. NaCl in a wide range of doses (0, 50, 100, 150, 200, 300 and 400 mM NaCl was added to MS medium and used for seed germination and seedling development of both tested species. For experiments with CNTs, MS medium supplemented with 100 mM NaCl and different concentrations of CNTs (MS with 100 mM NaCl and 50 μg/ml CNTs, MS with 100 mM NaCl and 100 μg/ml CNTs, MS with 100 mM NaCl and 200 μg/ml CNTs, MS with 100 mM NaCl and 500 μg/ml CNTs, MS with 100 mM NaCl and 1000 μg/ml CNTs) was prepared and sterilized seeds (sorghum and switchgrass) were then inoculated. Similarly, for experiments with graphene, MS medium supplemented with 100 mM NaCl and different concentrations of graphene (MS with 100 mM NaCl and 50 μg/ml graphene, MS with 100 mM NaCl and 100 μg/ml graphene, MS with 100 mM NaCl and 200 μg/ml graphene, MS with 100 mM NaCl and 500 μg/ml graphene, and MS with 100 mM NaCl and 1000 μg/ml graphene) were prepared and seeds of sorghum and switchgrass were inoculated. MS medium with no salt was used as a positive control whereas MS medium supplemented with 100 mM NaCl was used as a negative control (addition of 100 mM NaCl to the growth media significantly reduces the seed germination of switchgrass and plant growth of sorghum and switchgrass)

#### Incubation of bioenergy crops in controlled environmental conditions (greenhouse)

To investigate whether the observed biomass enhancement caused by application of CBNs to young seedlings will be reproduced for mature plants, we designed greenhouse experiments at the University of Arkansas at Little Rock. During the experimental period, the temperature was maintained at 32°C (day) and 26°C (night) with 16 hours day photoperiod. Seedlings of switchgrass and sorghum were transplanted in the pots (22 cm diameter and 18 cm height) containing autoclaved soil (sunshine Redi-earth soil from SUNGRO horticulture; www.sungro.com). Ten young seedlings (10 days old) for each treatment (control, 50 μg/ml CNTs, 200 μg/ml CNTs, and 50 μg/ml graphene, 200 μg/ml graphene) were transferred to the greenhouse and cultivated in the soil. 100 ml of nanoparticle solutions (50 mg/ml and 200 mg/ml CNTs) and (50 mg/ml and 200 mg/ml graphene) were applied to root system of one-month-old sorghum and switchgrass plants for three times over an interval of seven days. An equal amount of deionized water was used for irrigation to all plants during experimental conditions. For the control plants, the same amount of pure deionized water was applied to the soil around the root system. After 90 days of cultivation, plants were harvested and the phenotypic study was performed.

#### Statistical analysis

SPSS software (IBM SPSS Statistics 24) was used to perform One-way ANOVA. All data are expressed in average values ± SE (standard error). The repeated measure ANOVA for time-effect analysis as well as ANOVA and post-hoc analysis via Tukey test for treatment differences was used for data analysis. Statistical significance was determined by *p*<0.05 and *p* < .01 (* = *p* < .05 and ** = *p* < .01*)*.

#### RNA isolation cDNA synthesis and Real-time PCR

Total RNA was isolated from plants grown on pure MS medium, plants grown on MS medium supplemented with 100 mM of NaCl, and plants exposed to MS medium supplemented with 100 mM NaCl and range of concentrations of CNTs or graphene using RNeasy Plant Mini Kit (Qiagen Inc. Valencia, CA). The cDNA synthesis for all RNA samples was carried out using a SuperScript III First-Strand Synthesis System Kit (Invitrogen, Carlsbad, CA) with dT16- oligonucleotide primers according to the manufacturer’s protocol. The cDNA utilized for the Real-time PCR reaction using gene-specific primers (Table 1). Primers were designed by using NCBI database and IDT DNA web resource. Actin and 18S rRNA gene was used as an internal control.

**Table 1:** Primers used for Real-time PCR analysis of sorghum aquaporin.

Crops	Gene	Nucleotide sequence of primer	Product (bp)
**Sorghum**	*PIP 1;5*	F5'ACTGGATCTTCTGGGTTGGC-3' R 5'—TTAGTCGCGGCTCTTGAAGG-3'	98
**Sorghum**	*TIP 1;1*	F 5'-CCAACATCCTGGTCGGCG-3' R 5'-TACACCCACTGGTATCCCCA-3'	103

#### Potentiometric measurements of Na^+^ and Cl^-^ in salty solutions

To shed light on the mechanism of reduction of toxic symptoms in sorghum exposed to salt stress, we designed an experiment related to the evaluation of Na+ and Cl^-^ ion amounts in salty solutions supplemented with CNTs. Sodium selective electrode and chloride selective electrode sourced from Cole-Parmer (www.coleparmer.com) were used for our tests. It is well known that electrode potential directly correlates with the concentration of a specific ion in solutions investigated using an ion selective electrode [28]. Two separate experiments were performed for each electrode. First, using standard solutions, we constructed a standard curve as electrode potential (mV) versus concentration of salt ions (ppm) for each used electrode. Then, we measured the electrode potential of water and solutions with a range of NaCl concentrations (0; 0.5; 1; 1.5; 2; 2.5 mM) supplemented with 50 μg/ml of CNTs using sodium ion selective electrode. Finally, we measured the electrode potential of 1 mM NaCl solution and the same solution after adding the same volume of water and CNTs in the range of concentrations (50; 100; 200; 385 500; 1000 μg/ml).

#### Detection and quantification of multi-walled CNTs inside exposed crops using microwave induced heating (MIH) technique

CNT uptake by plant tissues was tested using the previously-published microwave induced heating (MIH) technique; the sample is exposed for a given time to microwaves (2.45 GHz) in a custom waveguide, and the temperature rise is correlated with local CNT content. Prior calibration curves for ΔT vs. multi-walled CNT loading (after 30 W, 10 sec exposure, and 50 W, 6 sec exposure) were utilized and adjusted as appropriate for these samples; the intercept is computed from the response of the control samples for each plant matrix. We established the CNT content of dry shoot samples (μg CNT) by comparing the temperature rise of the samples to the calibration curve. To estimate the average amount of multi-walled CNTs absorbed by different plant shoots (μg CNTs/mg plant) we divided the mass of CNTs in the sample by the mass of the shoot. Calibration curve for CNT detection is shown in S9 Fig

## Results

### Effect of CNTs and graphene on seed germination and development of sorghum and switchgrass seedlings

**Fig 2:**
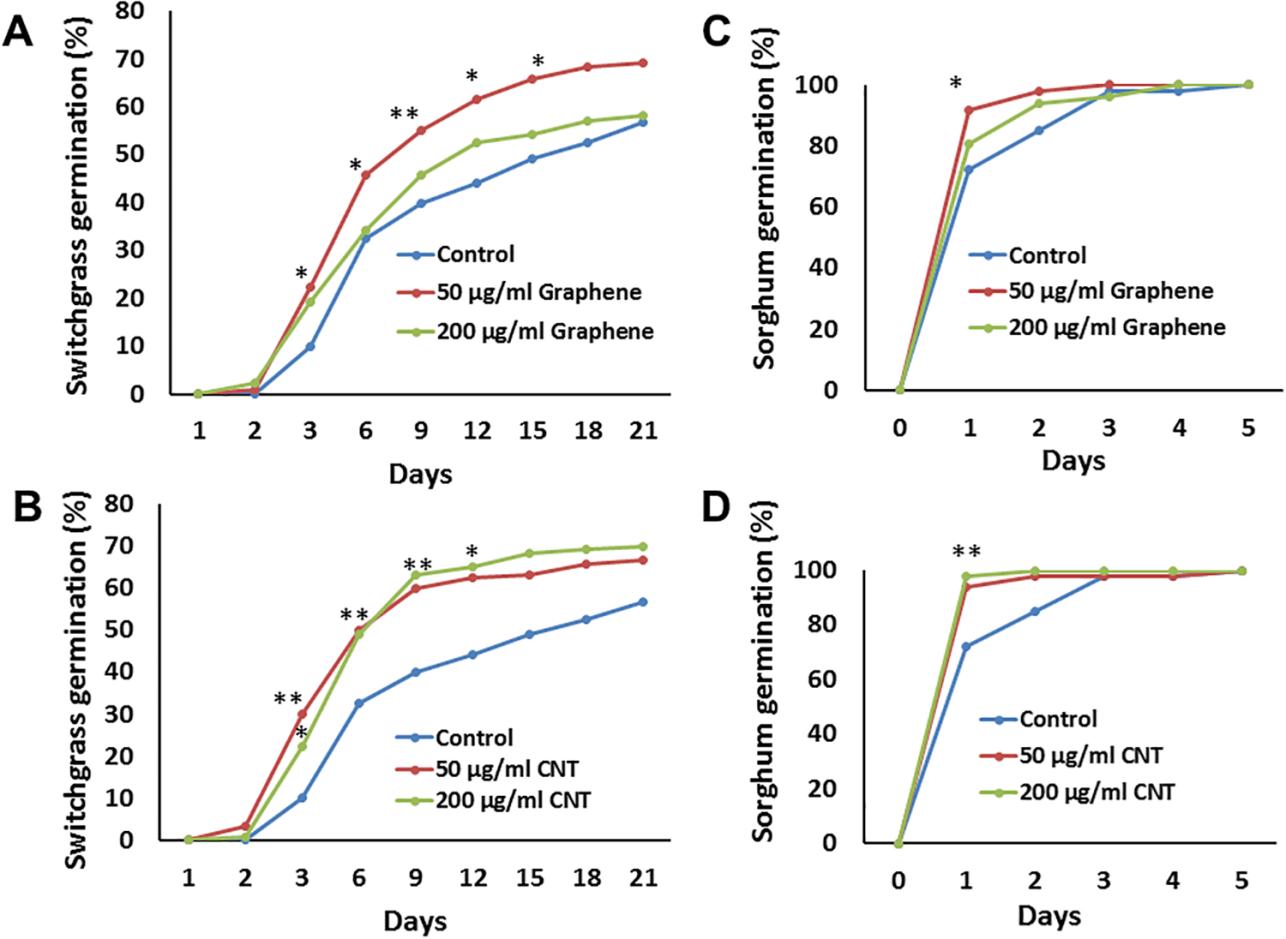
Activation of seed germination of switchgrass and sorghum by exposure to carbon-based nanomaterials (CBNs). The graphs show the germination rate of seeds of switchgrass (A, B) and sorghum (C, D) exposed to graphene (A, C) and multi-walled carbon nanotubes (B, D) by the addition of nanomaterials into the Murashige and Skoog growth medium. Seed germination rate is expressed in percentage. Each Magenta box contains ten sorghum seeds or 20 switchgrass seeds (n = 60 for each treatment of sorghum and n = 120 for each treatment for switchgrass). (* = *p* < .05 and ** = *p* < .01*)*.

The appearance of the first root was considered as a sign of germination. We concluded that the application of graphene and CNTs to seeds significantly enhanced the seed germination rate of switchgrass and sorghum (Fig 2). Both used CBNs (CNTs, graphene) in concentrations of 50 μg/ml and 200 μg/ml were effective stimulators for germination of both bioenergy species. The 50 μg/ml graphene concentration was more effective for stimulation of seed germination of switchgrass than 200 μg/ml (Fig 2A). The exposure of switchgrass seeds to 50 μg/ml graphene resulted in about 15% (p < .01) higher germination at day-9 whereas, exposure of seeds to 50 μg/ml CNTs increased the switchgrass germination by 20% (p < .01) as compared to untreated switchgrass at day-9. Similarly, the exposure to CNT (200 μg/ml) led to 19.16% (p < .05) higher seed germination as compared to control switchgrass seeds at day-15 of observation. The sorghum seed germination was enhanced by 21.89% (p < .05) and 19.78% at day-1 when exposed to 50 μg/ml CNTs and 50 μg/ml graphene respectively as compared to untreated sorghum seeds (Fig 2C and 2D).

**Fig 3:**
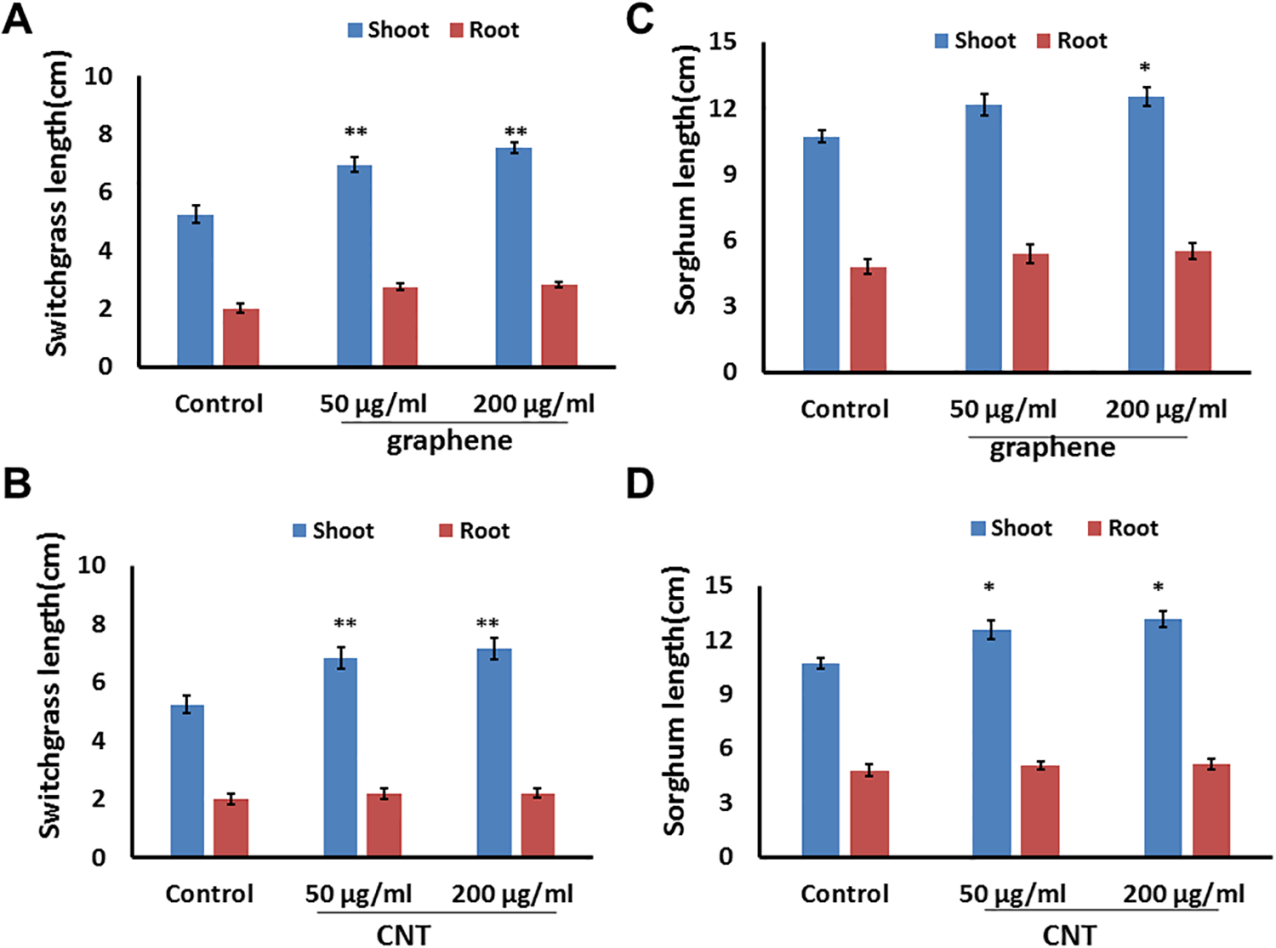
Growth enhancement of switchgrass (A, B) and sorghum (C, D) seedlings by exposure to carbon-based nanomaterials. Effects on growth of bioenergy crops by CNTs (B, D) graphene (A, C) added to growth medium. Measurements were performed on 10-day-old seedlings (n = 30 for both sorghum and switchgrass). (* = *p* < .05 and ** = *p* < .01*)*.

Detailed phenotypic analysis was performed on 10-day-old seedlings exposed to CBNs and unexposed to any nanomaterials. Seedlings growing on media supplemented with CNTs or graphene developed longer shoots with similar size of roots (Fig 3). Switchgrass shoot length was found to be significantly increased by the application of both types of CBNs at both tested concentration of CNTs (50 μg/ml and 200 μg/ml) (Fig 3A and 3B). Sorghum shoot length was significantly increased after exposure to 200 μg/ml of graphene or 50 μg/ml, 200 μg/ml of CNTs. As expected, the addition of CBNs to the media led to increasing of the total fresh biomass of exposed 10-day-old seedlings (S1 Fig). For example, exposure of switchgrass seedlings to CNTs led to 73.68% (p < .01) and 31.57% (p < .01) of shoot biomass at concentrations 50 μg/ml and 200 μg/ml respectively. The increase of total root biomass was also recorded for CNTs or graphene exposed switchgrass seedlings (S2 Fig).

### Effect of CNTs and graphene on sorghum and switchgrass growth in greenhouse conditions

**Fig 4:**
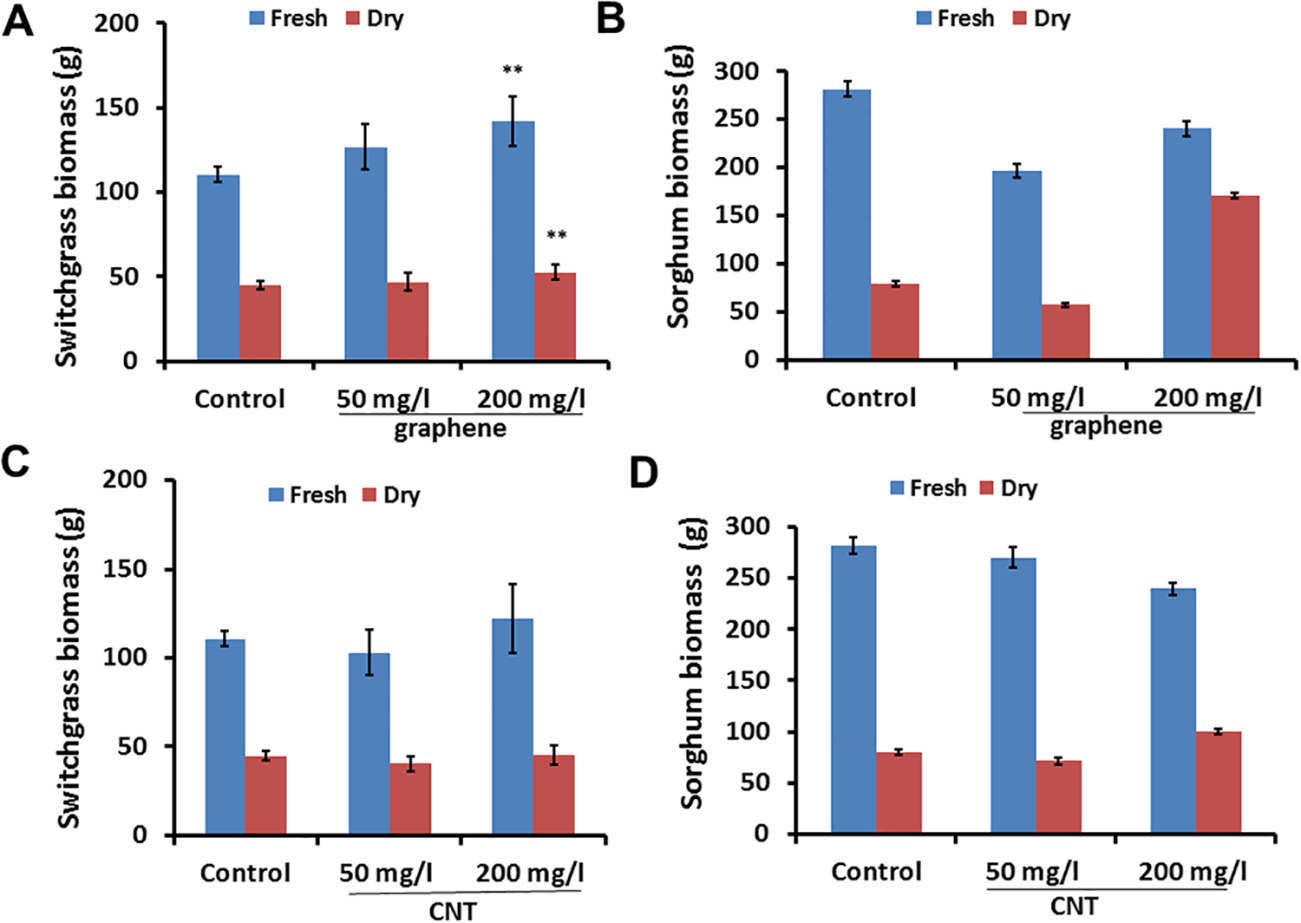
Effects of graphene (A, B) and CNTs (C, D) on shoot biomass production of bioenergy crops (sorghum, switchgrass) cultivated for 90 days in soil mix supplemented with CBNs (graphene, CNTs). (* = *p* < .05 and ** = *p* < .01*)*.

We have concluded that introduction of at least one type of CBNs into soil growth medium can lead to a significant increase of biomass production in bioenergy crops. Particularly, switchgrass plants treated with 200 mg/l of graphene added to soil produced 28.11% (p < .01) more fresh and 16.66% (p < .01) more dry shoot biomass compared to untreated (control) plants (Fig 4A). On the contrary, exposure of switchgrass plants to CNTs in soil did not lead to enhancement of biomass production (Fig 4C) of mature plants. Additionally, graphene positively affected reproductive system of bioenergy plants (S3 Fig) Thus, graphene (200 mg/l) stimulated the production of total fresh and dry grain head biomass of sorghum by 45.81% (p < .05) and 33.44% (p < .05) respectively. We also observed that the addition of graphene (50 μg/ml, 200 μg/ml) to soil mix led to a significant increase (66% and 47.14%) (p < .05) of seed heads per switchgrass plant (S3 Fig).

### Effect of CNTs and graphene on salt stress response of exposed sorghum and switchgrass plants

Toxicity of NaCl to young seedlings is presented in S4 Fig. We observed that the addition of NaCl to the medium was toxic for both bioenergy species and inhibited shoot and root growth of sorghum seedlings in a dose-dependent manner. The addition of 100 mM NaCl reduced the root length and shoot length of sorghum seedlings as compared with seedlings that were not exposed to NaCl (S4A and S4B Fig). Similarly, for switchgrass, the addition of 100 mM NaCl reduced seed germination and seedling growth (S4C and S4D Fig). 100 mM NaCl was selected as a suitable concentration for further experiments involving CBNs.

**Fig 5:**
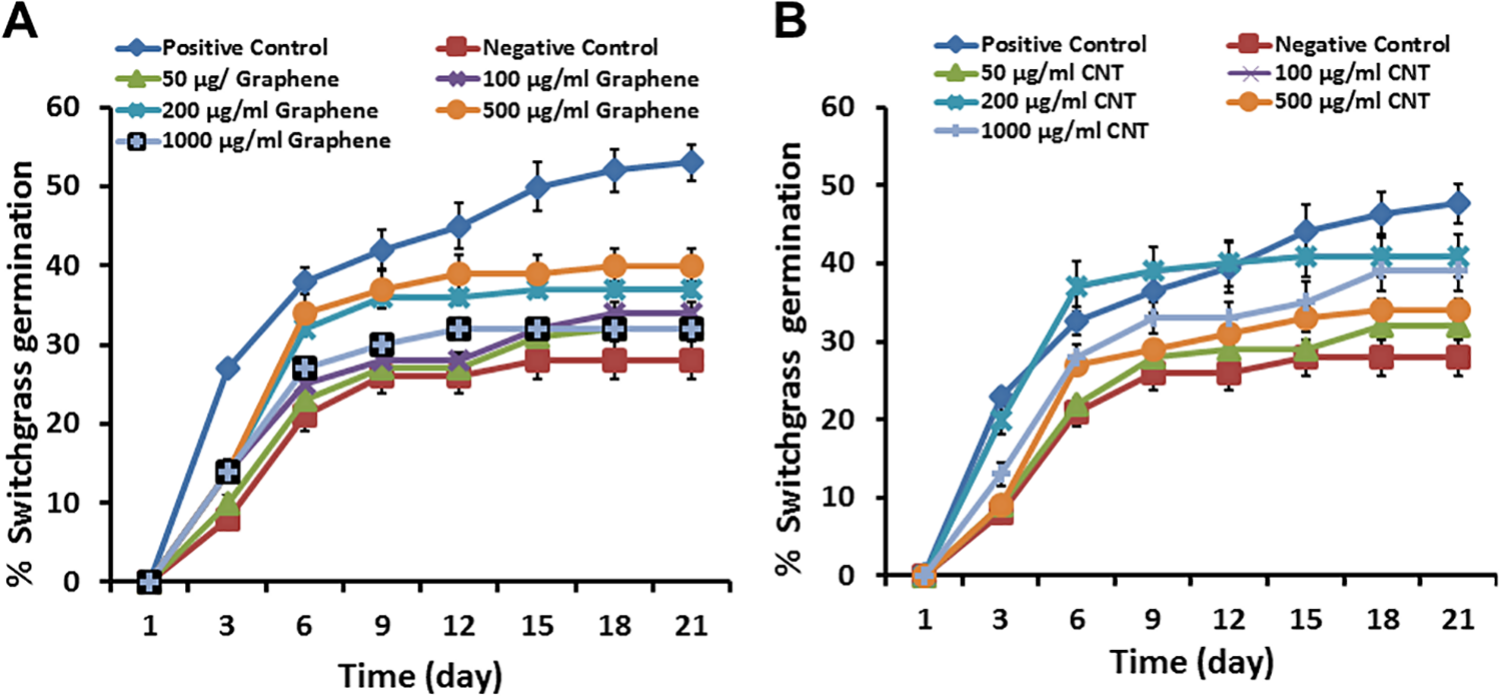
The addition of graphene (A) and multi-walled CNTs (B) can reduce the negative effect of NaCl on germination of switchgrass seeds. For positive control, seeds were placed on regular Murashige and Skoog medium. For negative control, seeds were placed on Murashige and Skoog medium (MS) supplemented with 100 mM NaCl. For treatment with CBNs, seeds were placed on MS medium supplemented with 100 mM NaCl and different concentrations of CNTs or graphene (50, 100, 200, 500, 1000 μg/ml).

We have concluded that introduction of CNTs or graphene in salty growth medium dramatically reduced inhibition of switchgrass seed germination caused by toxicity of NaCl (Fig 5). The highest germination rate of switchgrass seeds exposed to NaCl was observed at 500 μg/ml of graphene and 200 μg/ml of CNTs. Activated carbon (regular carbon size) was not effective for reversal or reduction of seed germination caused by the presence of sodium chloride in the growth medium (S5 Fig). The addition of CNTs and graphene to growth medium significantly reduced suppression of shoot and root length of switchgrass seedlings exposed to 100 mM NaCl during 21 days (S6 Fig).

**Fig 6:**
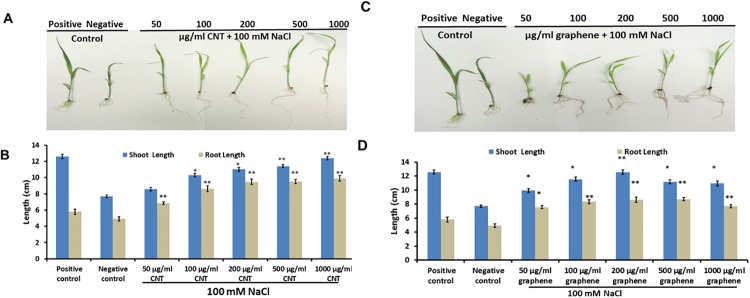
The addition of CNTs (A, B) and graphene (C, D) to growth medium reduce suppression of shoot and root length of 10-days old sorghum seedlings exposed to 100 mM NaCl. For positive control (P) seedlings were grown on regular Murashige and Skoog medium. For negative control (N) seedlings were grown on Murashige and Skoog medium (MS) supplemented with 100 mM NaCl. For treatment with CBNs, seedlings were grown on MS medium supplemented with 100 mM NaCl and different concentrations of CNTs or graphene (50, 100, 200, 500, 1000 μg/ml). (* = *p* < .05 and ** = *p* < .01*)*.

Due to the natural high germination speed of sorghum, we were not able to obtain reliable data of germination tests involving salt exposure to sorghum seeds and monitor the further development of sorghum seedlings exposed to NaCl and CBNs (Fig 6). As shown in Fig 6, shoot and root length of sorghum were dramatically reduced when NaCl (100 mM) was added to MS medium (negative control). However, this reduction was reversed when graphene or CNTs in concentrations of 100–1000 μg/ml were added to medium supplemented with sodium chloride.

### CBNs activate expression of water channel genes (aquaporins) in young seedlings of sorghum

**Fig 7:**
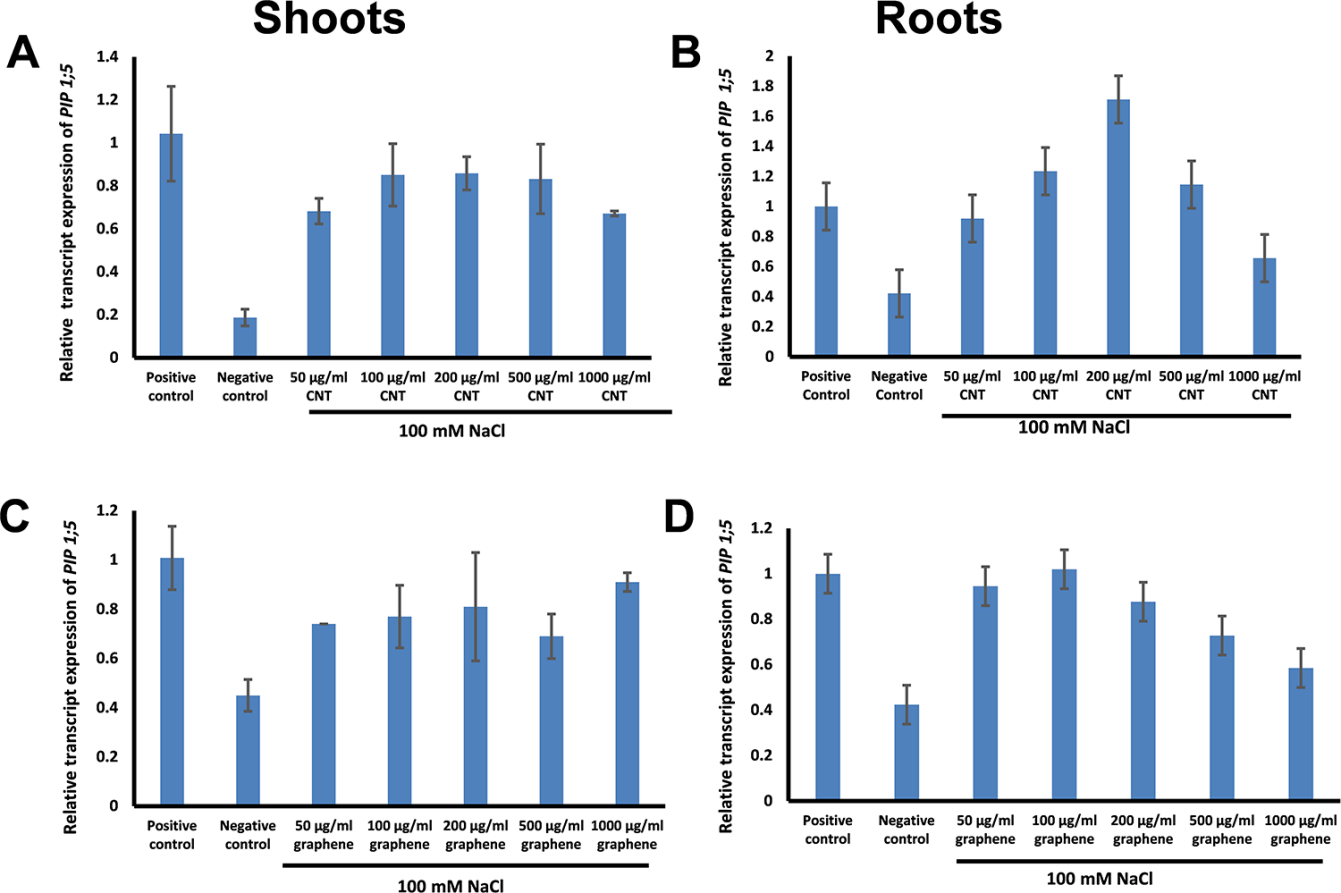
Real-time PCR analysis of expression of sorghum water channel gene (*PIP 1;5*) in 10 day-old sorghum shoots (A, C) and roots (B, D) grown in saline Murashige and Skoog medium (100 mM NaCl) supplemented with a wide range of CNTs concentrations (A, C) or graphene (B, D). For positive control, seedlings were grown on regular Murashige and Skoog medium. For negative control, seedlings were grown on Murashige and Skoog medium (MS) supplemented with 100 mM NaCl.

We have selected two common sorghum aquaporins (*PIP 1;5* and *TIP1;1*) that belong to different aquaporin families and performed real-time PCR analysis of both genes in sorghum roots and shoots exposed to NaCl (100 mM) with and without exposure to graphene and CNTs (Fig 7, S7 Fig). We found that expression of the *PIP 1;5* gene was reduced in shoots and roots of sorghum when NaCl was added to growth medium but was dramatically enhanced when the salty medium was also supplemented with graphene (Fig 7C and 7D) or CNTs (Fig 7A and 7B). Concentrations of 100–200 μg/ml for CNTs and 50–200 μg/ml for graphene were the most efficient for activation of *PIP 1;5* gene expression. A similar trend of expression activation in roots and shoots by graphene and CNTs was recorded for another sorghum aquaporin gene, *TIP 1;1* (S7A and S7B Fig).

### Measurements of electrode potentials of saline solutions supplemented with CNT using Na^+^ and Cl^-^ ion selective electrodes

**Fig 8:**
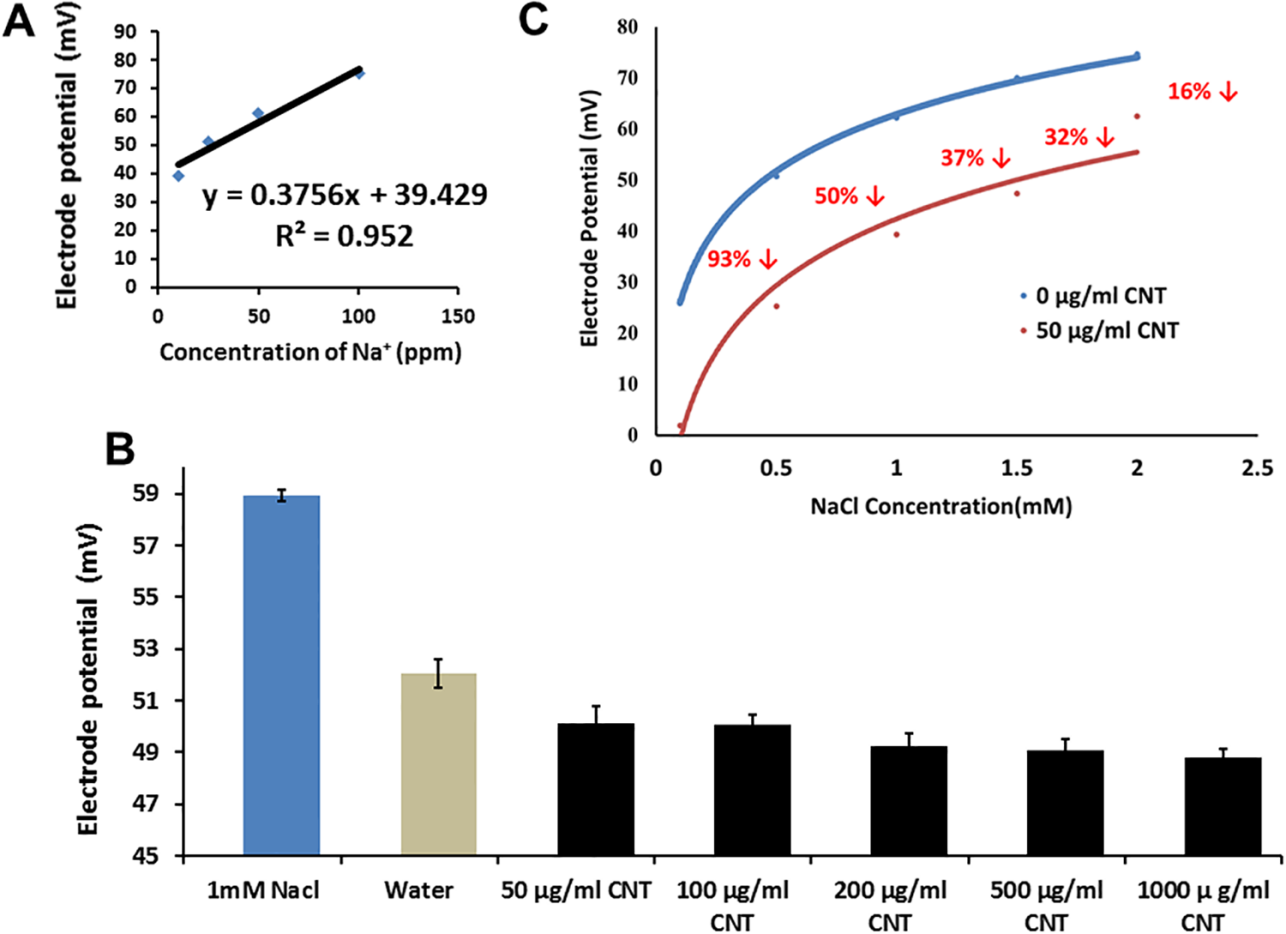
Measurements of electrode potential of saline solutions supplemented with CNTs using sodium ion selective electrode. A standard curve potential (mV) versus Na^+^ concentration (ppm) was performed using a sodium ion selective electrode (A), effects of a wide range of concentrations of CNTs on the electrode potential of 1 mM NaCl solutions (B) as well as effects of CNTs (50 μg/ml) on electrode potential of saline solutions with different concentrations of NaCl (C) were recorded. All the experiments were done in triplicate.

We noticed that the addition of water to 1 mM NaCl (dilution) resulted in an expected decrease in electrode potential (Fig 8C). However, when the same volume of CNT solution was added to 1 mM NaCl solution, the electrode potential of saline solution was further decreased. After data analysis, we have concluded that CNTs most likely can interact with sodium ions and probably absorb such Na^+^ ions. No significant effect on the potential of saline solutions was observed in tests utilizing the chloride selective electrode (S8A, S8B and S8C Fig).

### Quantification of CNTs absorbed by bioenergy crops using the microwave induced heating technique (MIH)

Dry shoot biomass of matured sorghum and switchgrass treated with CNTs and dry biomass of untreated (control) plants were used as samples for CNT detection. Calibration curve for CNT detection is shown in S9 Fig. As shown in Table 2 sorghum shoots of fully mature plants grown on soil supplemented with CNT accumulated 0.007±0.002 μg of CNTs per mg of dry sample. Switchgrass shoots grown on soil supplemented with CNTs accumulated 0.015±0.002 μg of CNTs per mg of dry sample. As expected, no CNT traces were found in dry samples of unexposed sorghum and switchgrass plants (Table 2).

**Table 2:** Microwave induced heating (MIH) quantification of MWCNTs inside the shoots of bioenergy crops grown in multi-walled CNT supplemented soil mix.

Sample	Power, Time	T_i_, °C	T_f_, °C	ΔT, °C	CNT mass, μg	μg CNT/mg sample	Avg. & STD
**Untreated sorghum (dry shoot biomass)**	30 W, 10 s	21	38	17	No CNT	-	-
		22	35	13			
	50 W, 6 s	22	38	16			
		22	39	17			
**Sorghum treated with CNTs added to soil (dry shoot biomass)**	30 W, 10 s	22	53	31	0.129	0.007	**Avg = 0.007** **STD = ±0.002**
		22	60	38	0.194	0.01	
		22	58	36	0.075	0.009	
	50 W, 6 s	22	55	33	0.119	0.006	
		22	52	30	0.097	0.005	
		22	52	30	0.097	0.005	
**Untreated switchgrass (dry shoot biomass)**	30 W, 10 s	22	44	22	No CNT		
		22	45	23			
	50 W, 6 s	22	52	30			
		22	49	27			
**Switchgrass treated with CNTs added to soil (dry shoot biomass)**	30 W, 10 s	22	81	59	0.535	0.017	**Avg = 0.015** **STD = ±0.002**
		22	79	57	0.517	0.016	
		22	79	57	0.517	0.016	
	50 W, 6 s	22	93	71	0.306	0.016	
		22	89	67	0.276	0.014	
		22	93	71	0.276	0.016	

## Discussion

The potential of carbon-based nanomaterials (CBNs) for agriculture was previously documented. It is known that seed germination, plant cell division, plant growth, and development can be enhanced by exposure of seeds, plant, or plant cells to different CBNs including carbon nanotubes, nanohorns, and graphene [11–17, 25, 29, 30]. Here, we documented that two types of CBNs (graphene, CNTs) can regulate seed germination and growth of major bioenergy crops such as sorghum and switchgrass. Both tested materials were efficient for stimulation of seed germination of sorghum and switchgrass and enhancement of the shoot length, root length, and shoot biomass of young seedlings germinated and grown on medium supplemented with graphene or CNTs. Such observations are in good correlation with results of similar experiments performed with tomato model plant [11, 12] and valuable crops (*Glycine max*, *Zea mays*, *Hordeum vulgare*) [15]. The enhanced production of total biomass (maximum production of dry matter per hectare) is the major desirable characteristic of the ideal bioenergy crops [31]. We have tested if the application of CBNs can lead to higher biomass production of mature sorghum and switchgrass plants. Greenhouse experiments revealed that application of graphene to the soil mix can increase production of total shoot biomass of switchgrass by 28.11%. The positive effect of graphene on the development of reproductive organs was recorded for both tested species. Activation of plant reproductive organ development in response to an application of CBNs is not a surprising observation and was described previously for tomato plants exposed to multi-walled carbon nanotubes [13]. Particularly, the introduction of CNTs to soil resulted in a significant increase of tomato flower number and tomato fruit production [12]. We recorded a similar effect for graphene introduced in soil on the reproductive system of sorghum and switchgrass (S3 Fig). It was unexpected that CNTs were not effective for growth regulation of mature bioenergy species. This observation confirmed that the type of CBNs used for growth regulation of a particular crop species has to be selected using a solid experimental base.

The novel effect of CBNs on plant stress response was also confirmed here. Thus, CBNs were effective for suppression of toxic symptoms of sorghum and switchgrass caused by salt stress imposed by the addition of NaCl in the growth medium. We have discovered that addition of graphene or CNTs to saline growth medium led to the restoration of seed germination rate and development of young seedlings. The mechanism of influence of CBNs to plant stress response can be based on the previously documented effect of CBNs on the plant transcriptome [12] and/or physical interaction of CBNs with toxic ions. Our data gave additional experimental evidence that both mechanisms can play a role in a documented enhancement of salt tolerance in CBN-exposed bioenergy species. Recently, we reported that CBNs can activate expression of water channel genes (aquaporins) at the gene [12], [15] and protein level [14] in different organs of several plant species exposed to CBNs. Aquaporins are a key player in plant–water relationships and are involved in the response of plants to environmental stress [32]. There are many indications that enhanced expression of aquaporins may lead to higher resistance of abiotic stress including salt stress [33]. For example, it was demonstrated that the overexpression of wheat aquaporin (*TaNIP*) in transgenic *Arabidopsis* resulted in a higher salt tolerance compared to wild-type plants [34]. Similarly, overexpression of another wheat aquaporin gene (*TaAQP8*) enhanced salt tolerance in transgenic tobacco [35]. Here, we are hypothesizing that stimulation of aquaporin expression by CBNs can play an important role in the observed tolerance of sorghum seedlings to the presence of NaCl (Fig 6). Sorghum is an appropriate model for expression analysis of aquaporins because 41 aquaporins were identified and classified in sorghum recently [36]. Thus, we showed here that expression of water channel genes (aquaporin) reduced by the presence of NaCl was restored as a result of CBNs (graphene or CNTs) addition into the medium. Taking into account that aquaporins play an important role in plant resistance to salt stress [34], we can suggest that CBNs may affect plant stress response by regulation of the expression of key genes of plant stress signal transduction. However, experiments involving ion-selective electrodes provided some confirmation that CBNs also can physically interact with positively charged ions and absorb toxic ions from saline solution. Other authors noticed that desalination [37]. The documented ability of CBNs to absorb salt from solutions used for plant growing practices can open new perspectives for agriculture. It is known that salt stress not only decreases the agricultural production of most crops, but also affects ecological balance of the area and physicochemical properties of the soil [38]. It was reported that more than 80 million hectors of irrigated land (about 40% of total irrigated land) have already been damaged by salt worldwide and salinization is expected to have devastating global effects, resulting in land loss up to 50% by the year 2050 [39]. It is well known that biomass productivity is responsive and negatively correlated to osmotic stress [40]. The overall productivity of bioenergy crops is decreasing every year due to salt stress. For example, exposure of sorghum plants to salt stress resulted in a reduction of plant growth and biomass productivity [41]. More recently, Stavridou et al., 2017 observed the gradual decline of dry biomass of *Miscanthus giganteus* in response to increasing salinity [23]. Thus, development of new technologies for desalination of water used for watering is a very desirable approach. Due to lack of proper freshwater for the irrigation of agricultural land, seawater desalination for sustaining agriculture production is being reported as an alternative water resource in Mediterranean countries [42]. For example, Silber et al., 2015 discussed the possibility to use desalinated water for irrigation of banana fields [43].

The amount of multi-walled CNTs absorbed by bioenergy crops exposed to multi-walled CNTs was determined by using the recently developed advanced analytical tool microwave induced heating (MIH) technique. This method was used recently for quantification of carbon-based nanomaterial with the tubular structure inside biological samples including a wide range of exposed plant organs [16, 18].

Here, we proved CBNs can be not only beneficial as plant growth enhancers for bioenergy crops but also play a positive role in the elimination of environmental stress symptoms of important crops including bioenergy species. Our discoveries can be used as a base for the development of new technologies focused on the introduction of salty or seawater solutions treated with CBNs in agricultural practice.

## Supporting information

S1 Fig Effect of graphene (A, C) and CNTs (B, D) added in growth medium on shoot biomass of seedlings of switchgrass (A, B) and sorghum (C, D). (TIF)

S2 Fig Effect of graphene (A, C) and CNTs (B, D) added in growth medium on root biomass of seedlings of switchgrass (A, B) and sorghum (C, D). (TIF)

S3 Fig Effect of graphene added to soil on grain head biomass (A) of sorghum and number of seed head of switchgrass (B). (TIF)

S4 Fig Effects of NaCl on the development of 10-days-old sorghum seedlings (A, B) and 21-days-old switchgrass seedlings (C, D). (TIF)

S5 Fig Effect of activated carbon on germination rate of switchgrass seeds exposed to 100 mM NaCl. For positive control, seeds were placed on regular Murashige and Skoog medium. For negative control, seeds were placed on Murashige and Skoog medium (MS) supplemented with 100 mM NaCl. For treatment with activated carbon, seeds were placed on MS medium supplemented with 100 mM NaCl and different concentrations of activated carbon (50, 100, 200, 500, 1000 μg/ml).
(TIF)

S6 Fig Reduction of the toxic effect of NaCl by the addition of CNTs (A, B) or graphene (C, D) to saline growth medium used for cultivation of switchgrass seedlings (21-days-old). For positive control, seedlings were grown on regular Murashige and Skoog medium. For negative control, seedlings were grown on Murashige and Skoog medium (MS) supplemented with 100 mM NaCl. For treatment with CBNs, seedlings were grown on MS medium supplemented with 100 mM NaCl and different concentrations of CNTs or graphene (50, 100, 200, 500, 1000 μg/ml).
(TIF)

S7 Fig Real-time PCR analysis of expression of sorghum water channel gene (*TIP 1;1*) in 10 days-old sorghum roots (A, B) and shoots (C, D) at the saline environment (100 mM NaCl) supplemented with a wide range of concentrations of CNTs (A, C) or graphene (B,D). For positive control, seedlings were grown on regular Murashige and Skoog medium. For negative control, seedlings were grown on Murashige and Skoog medium (MS) supplemented with 100 mM NaCl. For treatment with CBNs, seedlings were grown on MS medium supplemented with 100 mM NaCl and different concentrations of CNTs or graphene (50, 100, 200, 500, 1000 μg/ml).
(TIF)

S8 Fig Measurements of electrode potential of saline solutions supplemented with CNTs using chloride ion selective electrode. A standard curve as mV versus ppm of Cl¯ concentration was performed using chloride ion selective electrode (A), effects of different concentrations of CNTs (50–1000 μg/ml) on Cl¯ electrode potential of saline solutions with NaCl (B) as well as effects of different volume of CNTs (5 to 20 ml with final concentration of CNTs 50 μg/ml) on electrode potential of 2 mM NaCl solutions were recorded. All the experiments were done in triplicate.
(TIF)

S9 Fig Calibration curve for the detection of CNTs inside the shoots of CNTs-exposed bioenergy crops. (TIF)
