# Supplementary figures and images for: Effects of carbon-based nanomaterials on seed germination, biomass accumulation and salt stress response of bioenergy crops
Source: PLoS One. 2018 Aug 28;13(8):e0202274. doi: 10.1371/journal.pone.0202274 (PMC6112629; doi:10.1371/journal.pone.0202274)

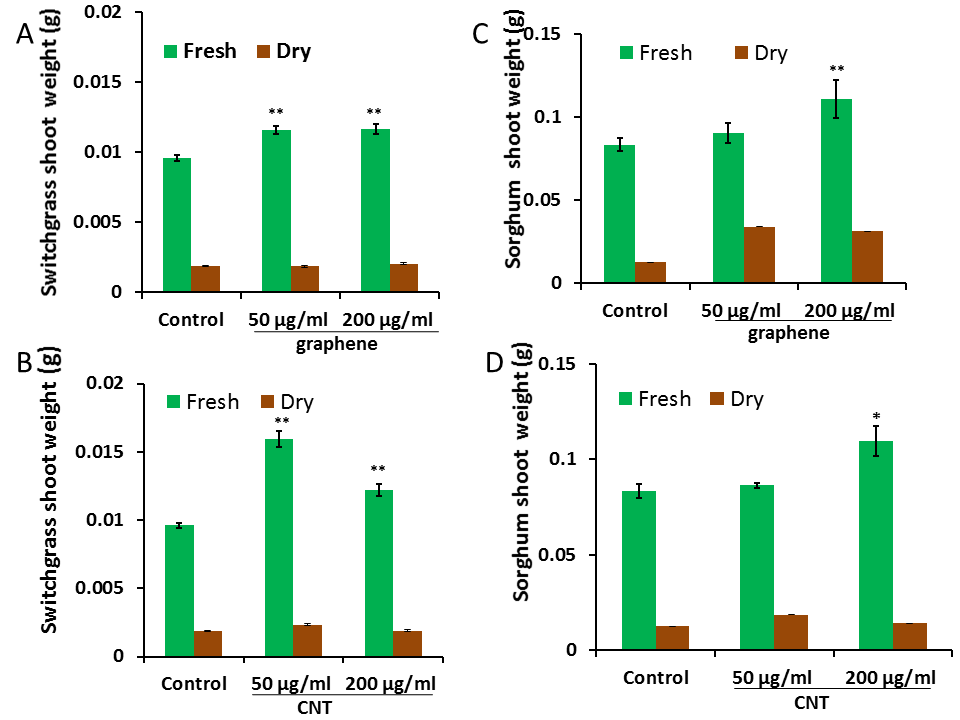

Supplement: S1 Fig — (TIF) [file pone.0202274.s001.tif]

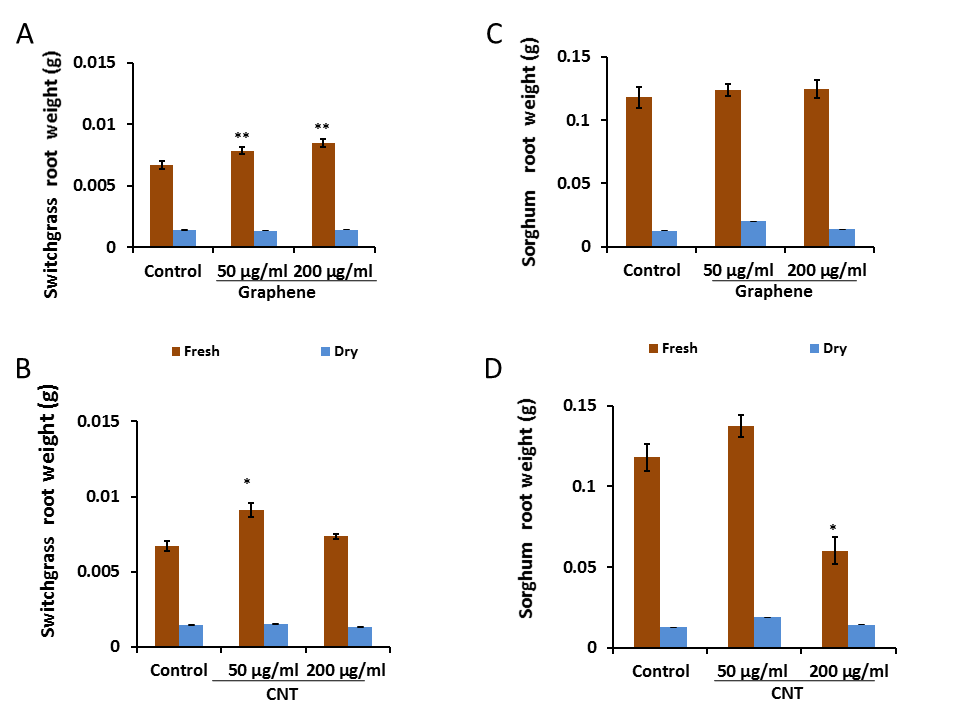

Supplement: S2 Fig — (TIF) [file pone.0202274.s002.tif]

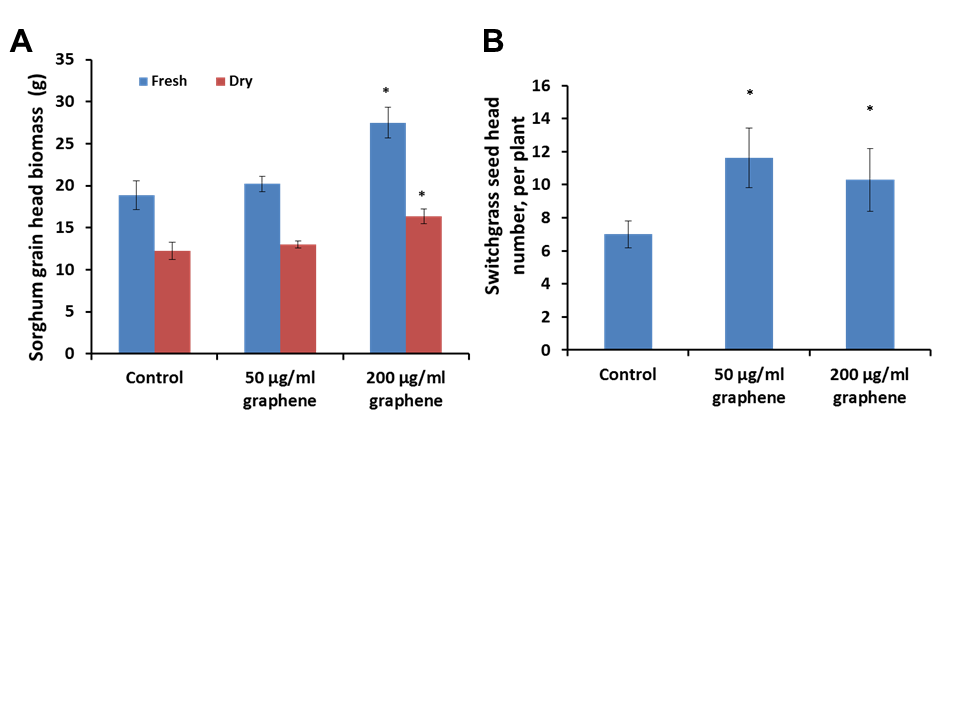

Supplement: S3 Fig — (TIF) [file pone.0202274.s003.tif]

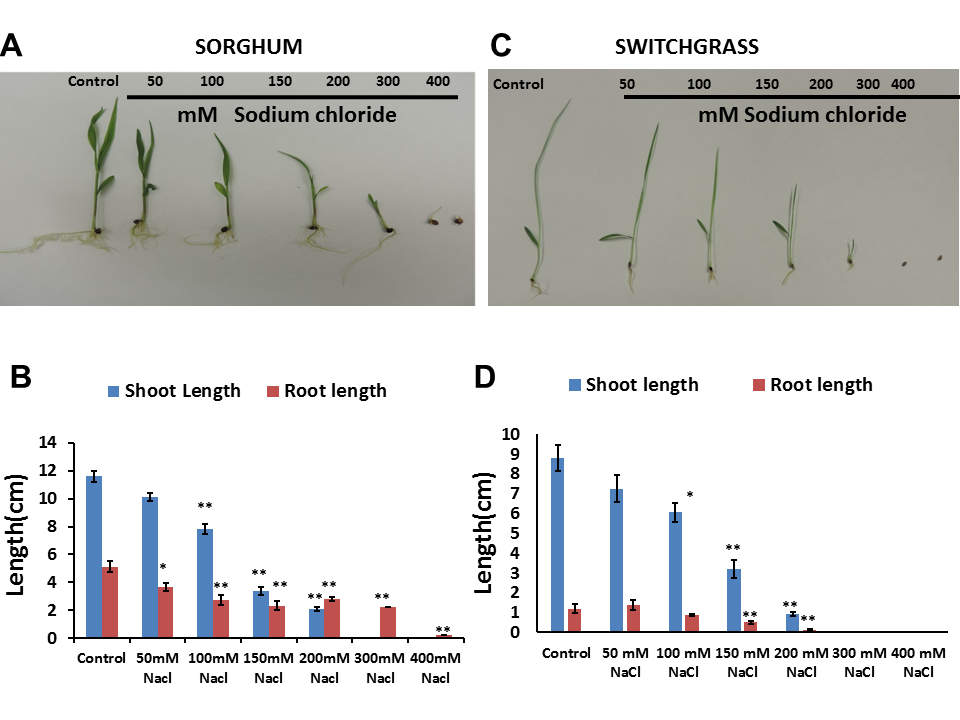

Supplement: S4 Fig — (TIF) [file pone.0202274.s004.tif]

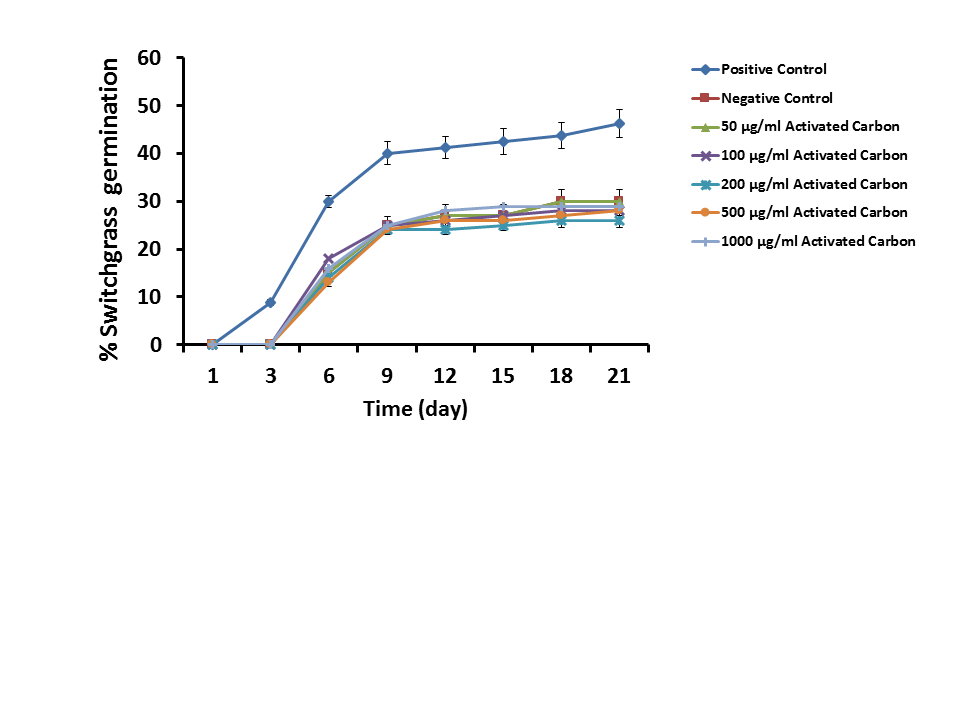

Supplement: S5 Fig — For positive control, seeds were placed on regular Murashige and Skoog medium. For negative control, seeds were placed on Murashige and Skoog medium (MS) supplemented with 100 mM NaCl. For treatment with activated carbon, seeds were placed on MS medium supplemented with 100 mM NaCl and different concentrations of activated carbon (50, 100, 200, 500, 1000 μg/ml). (TIF) [file pone.0202274.s005.tif]

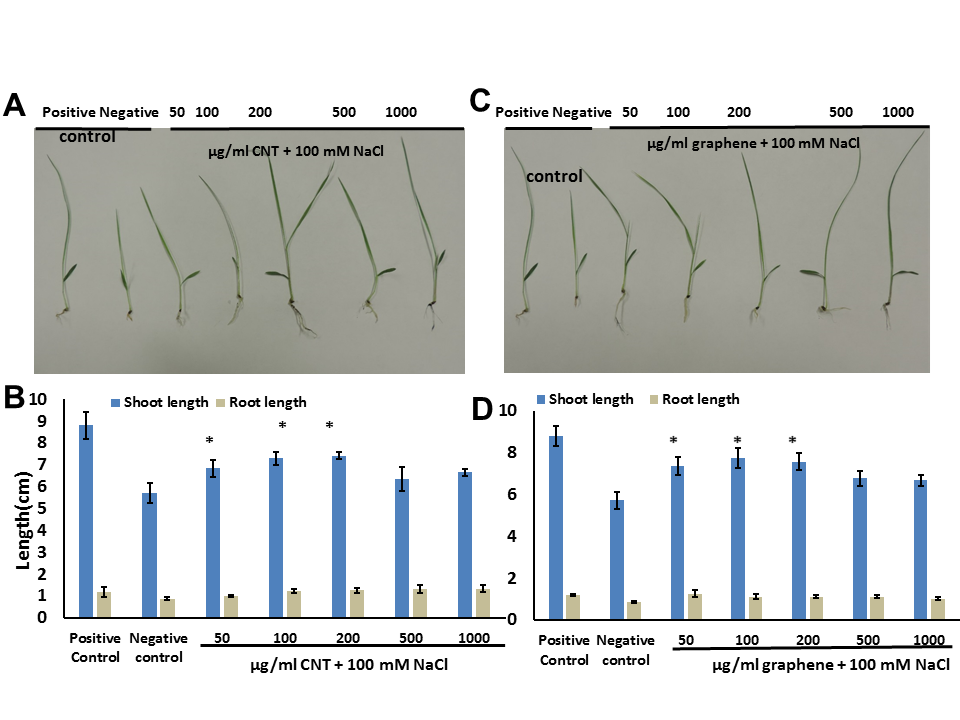

Supplement: S6 Fig — For positive control, seedlings were grown on regular Murashige and Skoog medium. For negative control, seedlings were grown on Murashige and Skoog medium (MS) supplemented with 100 mM NaCl. For treatment with CBNs, seedlings were grown on MS medium supplemented with 100 mM NaCl and different concentrations of CNTs or graphene (50, 100, 200, 500, 1000 μg/ml). (TIF) [file pone.0202274.s006.tif]

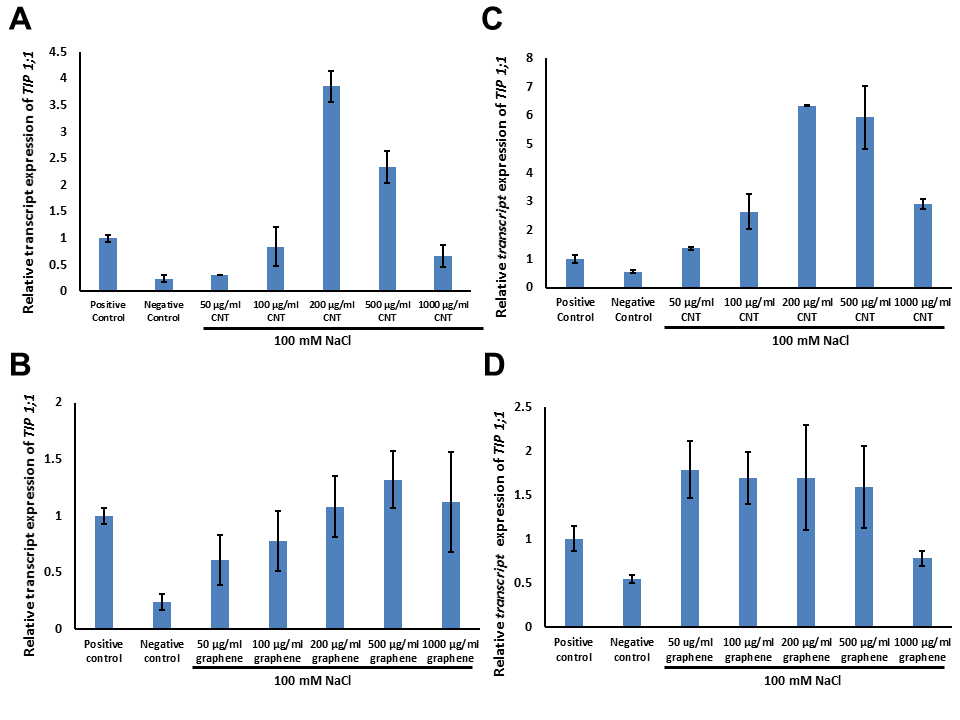

Supplement: S7 Fig — For positive control, seedlings were grown on regular Murashige and Skoog medium. For negative control, seedlings were grown on Murashige and Skoog medium (MS) supplemented with 100 mM NaCl. For treatment with CBNs, seedlings were grown on MS medium supplemented with 100 mM NaCl and different concentrations of CNTs or graphene (50, 100, 200, 500, 1000 μg/ml). (TIF) [file pone.0202274.s007.tif]

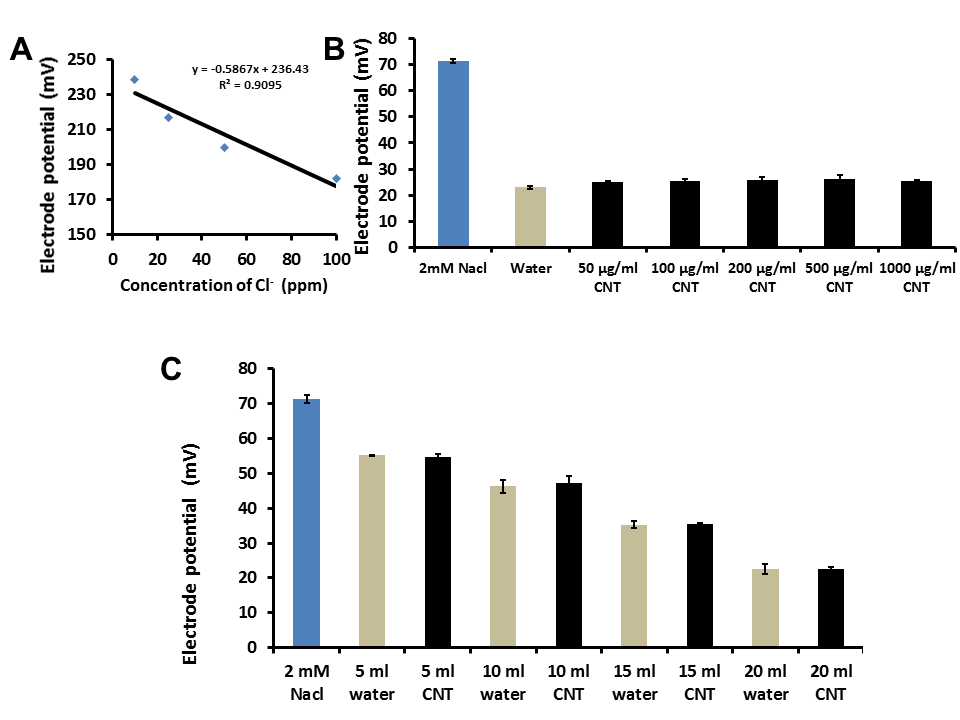

Supplement: S8 Fig — A standard curve as mV versus ppm of Cl¯ concentration was performed using chloride ion selective electrode (A), effects of different concentrations of CNTs (50–1000 μg/ml) on Cl¯ electrode potential of saline solutions with NaCl (B) as well as effects of different volume of CNTs (5 to 20 ml with final concentration of CNTs 50 μg/ml) on electrode potential of 2 mM NaCl solutions were recorded. All the experiments were done in triplicate. (TIF) [file pone.0202274.s008.tif]

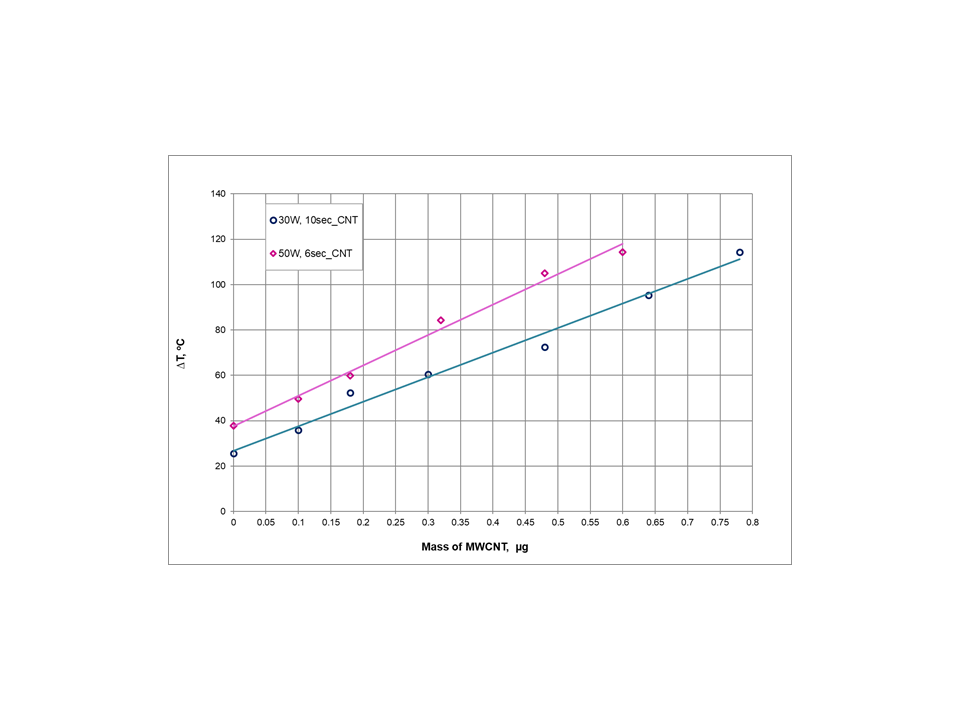

Supplement: S9 Fig — (TIF) [file pone.0202274.s009.tif]
